# Transmembrane mucins in lung adenocarcinoma: understanding of current molecular mechanisms and clinical applications

**DOI:** 10.1038/s41420-025-02455-3

**Published:** 2025-04-10

**Authors:** Xiaoqing Li, Ying Chen, Rui Lan, Peng Liu, Kai Xiong, Hetai Teng, Lili Tao, Shan Yu, Guiping Han

**Affiliations:** 1 Department of Pathology, Second Affiliated Hospital of Harbin Medical University, Harbin, China; 2 Laboratory of Medical Genetics, Harbin Medical University, Harbin, China; 3 Department of Statistic, Inner Mongolia Forestry General Hospital, Yakeshi, China; 4 Department of General Surgery, Inner Mongolia Forestry General Hospital, Yakeshi, China; 5 Department of Pathology, Peking University, Shenzhen Hospital, Shenzhen, China; 6 Heilongjiang Mental Hospital, Harbin, China

**Keywords:** Non-small-cell lung cancer, Tumour biomarkers

## Abstract

The mucin family is a group of highly glycosylated macromolecules widely present in human epithelial cells and with subtypes of secreted and membrane-associated forms. The membrane-associated mucins, known as transmembrane mucins, are not only involved in the formation of mucus barrier but also regulate cell signal transduction in physiological and pathological status. Transmembrane mucins could contribute to lung adenocarcinoma (LUAD) proliferation, apoptosis, angiogenesis, invasion, and metastasis, and remodel the immune microenvironment involved in immune escape. Furthermore, transmembrane mucins have been explored as potential LUAD indicators for diagnosis and prognosis. The development of targeted therapy and immunotherapeutic drugs targeting transmembrane mucins has also provided broad application prospects for clinic. In the following review, we summarize the characteristic structures of diverse transmembrane mucins, regulatory roles in promoting the progression of LUAD, and the current situation of diagnosis, prognosis, and therapeutic strategies based on transmembrane mucins.

## Facts

- Transmembrane mucins are glycoproteins composed of mucopolysaccharide. These proteins not only form a protective layer on the cell membrane but also participate in signal transduction and cellular response to environmental changes.
- The abnormal expression of transmembrane mucin is closely related to the occurrence and development of a variety of tumors. MUC1, MUC4, and MUC16 are involved in regulating the cancer hallmarks of lung adenocarcinoma and promoting its progression.
- Mucin-targeted medications and immunotherapy (vaccine, chimeric antigen receptor (CAR)-T cell therapy, etc.) are the two main categories of mucin-based medicines used in clinical anticancer treatment at the moment.

## Open questions

- How to improve the specificity and sensitivity of transmembrane mucin as an early diagnosis of lung adenocarcinoma?
- What is the significance of exploring the special epitopes formed by abnormal glycosylation of transmembrane mucin for the treatment of lung adenocarcinoma?
- In addition to immunotherapy and targeted therapies, what other therapeutic approaches can be developed that exploit transmembrane mucin for the treatment of lung adenocarcinoma?

## Introduction

The mucin family is classified into two groups: transmembrane mucins and secretory mucins [1]. Transmembrane mucins are typically a group of glycoproteins with a molecular weight greater than 200 kDa. They are mainly composed of mucopolysaccharides, including MUC1, MUC3A, MUC3B, MUC4, MUC12, MUC13, MUC14, MUC15, MUC16, MUC17, MUC20, MUC21 and MUC22 in humans named by order of discovery. Transmembrane mucins are known to activate intracellular signal transduction and regulate downstream gene expression through interactions with receptor tyrosine kinases (RTKs) by their structural regions, such as epidermal growth factor-like (EGF) domains or cytoplasmic tails [2]. Transmembrane mucins, synthesized by epithelial cells, widely exist in human organs and tissues, such as the respiratory system, digestive tract, mammary gland, and tear film of ocular [3–6]. Particularly, the respiratory system possesses the most abundant kinds of transmembrane mucins such as MUC1, MUC4, and MUC16, forming mucin barrier for protecting against infectious from microorganisms [7]. More than physical defense, transmembrane mucins participate in regulating epithelium homeostasis and pathological processes in benign and malignant pulmonary diseases by aberrant expressions or modifications. For instance, the transmembrane mucins help the cilia to move bacteria out of the airway in healthy people [8]. In the state of airway inflammation, MUC1 plays an anti-infective role but promotes the fibrosis process in infection-related pulmonary fibrosis [9]. In tumor status, abnormal upregulation of MUC1 promotes angiogenesis in non-small cell lung cancer (NSCLC) [10]. Besides, MUC16 overexpression affects different biological behaviors of NSCLC cells, including increasing proliferation, invasion, and their resistance to cisplatin [11]. Accumulating evidences highlight the importance of transmembrane mucins in physiology and pathology of the respiratory system, especially the influence of NSCLC. The following strategies could be considered to improve the specificity and sensitivity of transmembrane mucin as an early diagnosis of LUAD, such as combined detection of multiple markers in ctDNA; targeting aberrant glycosylation of MUC1 and development of nanoprobes detection technologies.

## Structure, biofunctions, and location of transmembrane mucins

### Structure and structure-based biofunctions

**Fig. 1:**
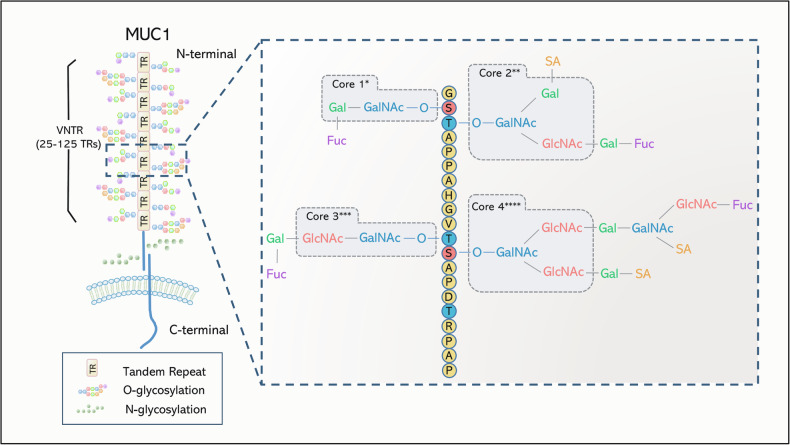
The biological structure of MUC1. The VNTR in MUC1-N consists of 20 amino acid repeats that are extensively O-glycosylated at serine and threonine residues. The structure of mucin O-glycan can be divided into four main core structures. Gal, GalNAc, Fuc, and SA residues are further added to them to extend the branches. ^*^Core 1 is formed by the transfer of Gal to an O-linked GalNAc residue. ^**^Core 2 is the second branch of a GalNAc residue that is formed by adding another GlcNAc to Core 1. ^***^Core 3 was formed by adding GlcNAc to the GalNAc section. ^****^Core 4 is a second branch of the GalNAc residue by adding another GlcNAc to Core 3.

As type I membrane proteins, transmembrane mucins are composed of three parts: an extensive O-glycosylation N-terminal extracellular region, a transmembrane domain, and a short C-terminal cytoplasmic tail. Transmembrane mucins with known structures have dissimilar numbers and types of functional domains in extracellular regions, including the presence of proline, threonine, and serine residues (PTS) domain, epidermal growth factor (EGF) like domain, sperm protein, enterokinase, and agrin (SEA) domain. Of these domains, the PTS domain, not conserved at the genomic level, is a main feature of transmembrane mucins. It forms a protein core of a variable number of tandem repeats (VNTR) connected with GalNAc on the serine and threonine residues, shaping an O-linked glycosylation about 100 nm [12, 13]. The tandem repeats in MUC1 (ranging from 25 to 125 repeats) confer molecular diversity through variable glycosylation patterns and are associated with increased susceptibility to lung adenocarcinoma (LUAD) and poorer prognosis. Similar associations have been observed for MUC4 [14, 15]. The MUC1 molecular structure is shown in Fig. 1. The biologically related features of glycocalyx and its component O-glycosylation are known as below: (1) The glycocalyx is hydrophilic, thus promoting hydration and lubrication of the surface epithelium. (2) The glycocalyx extends further from the cell surface than other extracellular receptors, assisting epithelium in disrupting the adhesion of harmful cells, pathogens, and strengthening physical defense [3, 12]. (3) The O-glycosylation serves as a recognition site for other proteins or cytokines, such as binding to the lectin family of the glycan-binding proteins on immune cells and modulating immune response to inflammation of human airway epitheliums [16]. (4) The O-glycosylation can be modified (sialylation, fucosylation, or sulphation), so-called abnormal glycosylation in cancer, in order to alter biochemical functions and provide potential ligands for receptors on the cell surface, assisting in tumor migration and immunosuppression [7]. As proof, MUC1 in cancer is proven to express a variety of simple and short sugar chain antigens such as Tn, sialyl-Tn, and sialyl-Lewis-X, emerging targets for cancer diagnosis and immunotherapy. A research depicted that LUAD patients expressing high levels of MUC1-Tn had a lower 5-year survival rate, and these cancer cells hold more mesenchymal features [17]. Sialyl-Tn of MUC1 could be recognized by monoclonal IgG antibody B72.3 [18]. (5) The length of glycocalyx affects tumor cells' aggressiveness. The longer the glycocalyx length, the more easily the tumor cells metastasize. The physical rationale is perhaps that the large glycocalyx drives integrin aggregation through the kinetic funnel so as to enhance levels of integrin-FAK and AKT signaling [19].

**Fig. 2:**
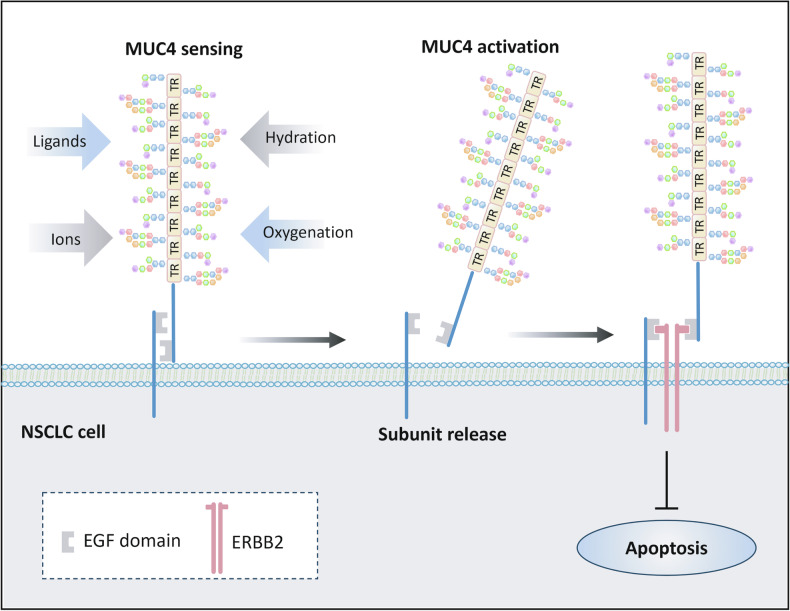
The process of activating the EGF domain of MUC4 and binding ERBB2. MUC4 in the resting state senses changes in the extracellular environment, including extracellular ligands, ions concentration, oxygenation, and hydration. Then the subunit release and the EGF domains are exposed. The exposed EGF domains attach to ERBB2, which would prevent NSCLC cells from apoptosis.

The EGF-like domain, located on the extracellular subunit, is 30-40 residue-long with a well evolutionary conservation [12]. In the resting state, the six cysteine residues in the EGF-like domain form three different disulfide bonds. It is considered that EGF domains on mucins serve a function of receptor–ligand interactions [20]. Based on the research of MUC4 molecular structure, when MUC4 is activated by extracellular stimulating factors (such as binding to lectins or adhesion molecules, ionic concentration, oxygenation, and hydration), the extracellular subunits undergo lysis and are released. In this way, the EGF-like ligands located in both extracellular and transmembrane subunits could be exposed at the same time [20]. The exposed EGF-like ligands could bind to membrane protein receptors epidermal growth factor receptor 2(ERBB2), leading to the suppression of apoptosis in NSCLC [21] (Fig. 2).

The SEA domain is a highly evolutionary conserved domain of about 120 residues, named after the three proteins originally identified (Sperm protein, Enterokinase, and Agrin) [12, 20]. According to current research, the main role of SEA domain is to act as the site for proteolytic cleavage [12]. MUC1, MUC3, MUC16, and MUC17 are cleaved in the SEA domain during post-translational processing into two subunits that stay together as they travel from the endoplasmic reticulum (ER) to the Golgi complex and ultimately to the cell membrane [22]. Besides, the disruption of SEA domain allows mucins to shed first to protect cells from rupture if epithelium cells suffer from mechanical force. In addition, bacteria adhesion with extracellular region of MUC1, MUC3, MUC12 induces mucin cleavage and shedding extracellular submits in the SEA domain, finally triggering signal cascades submits. For instance, Pseudomonas aeruginosa binds to MUC1 extracellular region and causes tyrosine phosphorylation of its intracellular domain [23–26].

The cytoplasmic tails (CT) of transmembrane mucins vary widely in sequence, with a length of less than 100 amino acids [12]. It’s known that the phosphorylated MUC1-CT preferentially and competitively binds to β-catenin with the WNT signaling transcription factor TCF/LEF, thus regulating transcription of genes involved in epithelial-mesenchymal transition [27]. Helicobacter Pylori infection also could trigger the MUC1-CT binds to IKKγ, blocking the activation of NF-κB and reducing the production of IL-8, which results in relieving the inflammatory response and protecting the gastric mucosa [28]. MUC1-CT also interacts with toll-like receptors (TLRs) to participate in immunoregulation. In the epithelial cells of the airway, epidermal growth factor receptor (EGFR) phosphorylates MUC1-CT, resulting in MUC1-CT binding to TLR5 competitively, inhibiting NF-κB and MyD88 dependency pathway, ultimately suppressing the release of inflammatory mediators and cytokines [29]. The MUC13-CT is verified to accelerate LUAD progression via aberrant activation of the ERK/JNK/p38 signaling pathway [30].

### Distribution in human tissues

Transmembrane mucins are widely expressed across the tissues of human body. It’s known that MUC1, MUC4, MUC11, MUC13, and MUC16 are majorly localized in bronchial surface epithelium, MUC1 is expressed in bronchial epithelium, collecting ducts, bronchioles, and submucosal gland. MUC4 is located in goblet cells, collecting ducts, and type II alveolar epithelial cells [31, 32]. MUC1, MUC3, MUC4, MUC12, and MUC13 have been established to be expressed in the gastrointestinal tract [33–35]. MUC1, MUC4, MUC13, MUC15, MUC16, and MUC17 are present in the conjunctival epithelium of human eyes [36]. Besides, MUC1 and MUC4 are disposed in the lactating epithelial cells. MUC1 is discovered not only in oral mucosal cells but also in major and minor salivary glands [37]. MUC4 is also found in body fluids such as saliva and ear fluid [7, 38]. MUC16 is preferentially expressed in the female genital tract surface epithelia and mesothelial cells [35, 39]. The unique mucin detected in the fallopian tube is MUC1[36].

### Characterization on the cell surface

The three-dimensional morphology of epithelial cells is not random or uniform, but exhibits axiality (apical-basal axiality), that is, polarity. The transmembrane mucins are located in apical side of cell surface (such as airway and gastrointestinal epithelial cells) facing the external environment owing to the polarized transport [38]. One traffic way is that the transmembrane mucins transport from the endoplasmic reticulum to the Golgi complex, packaged into vesicles then targeted traffic to apical cell membrane for anchor and fusion; the other way is endocytosis mediated by clathrin, caveolin or other endocytic pathways then the vesicles fuse with early endosomes of which could select the inside cargo, recycled by trans-Golgi and late/recycling endosome, or directly degraded by lysosomes [22]. It has been established that actin-disrupting drugs could destroy the apical localization of MUC1, which indicates that the interaction with actin cytoskeleton may be involved in MUC1 targeted localization. Besides, researchers validated two discrete signals guiding MUC1 apical localization: one was the Cys-Gln-Cys (CQC) motif located in transmembrane domain close to the cytoplastic side, the other one was located in extracellular domain suspected to mediate fusion [37]. The C-terminal PDZ domain of MUC17 was confirmed to bind to the scaffold protein PDZK1, which helped MUC17 locate at the apical membrane of small intestine epithelium cells stably [40].

The loss of polarity in cancer cells is partly due to the dysfunction of the following three polarity complexes, including the Crumbs complex, the Par complex, and the Scribble complex, which lead to the destruction of cell-cell adhesion structures and cytoskeletal rearrangement [41]. MUC1 and MUC4 overexpression exacerbate polarity loss by disrupting tight junctions via ERBB2-mediated Par complex inhibition [42, 43]. Due to disordered distribution, transmembrane mucins could cause unexpected signals by interacting with effector proteins on other side of cell surface. The aberrant distributed transmembrane mucins were supposed to combine with receptor tyrosine kinases (RTK), which are only located in basal and lateral membranes. For example, in the state of loss of polarity, MUC1 was revealed to be distributed anywhere on the cancer cell membrane, thus interacting with EGFR by galectin-3 and then activating the PI3K/AKT pathway, which was associated with increased angiogenesis in NSCLC [10, 44].

The expression pattern of transmembrane mucins extending from the apical to the whole surface of the cell membrane is known as depolarization. For instance, in lung cancer cells, the aberrant redistributed MUC1 with long carbohydrate chains could shield the smaller cell adhesion molecules (or ligands to adhesion molecules). In this way, cancer cells would be prohibited from interacting with adjacent cells, thus becoming more aggressive and metastatic [45, 46].

### Subcellular localization

It is also reported that the C-terminal of MUC1, MUC13, and MUC16 has been detected in nucleus [27]. The importin-β and nucleoporin 62 assisted in MUC1 C-terminal subunit (MUC1-C) translocation into nucleus and were located in nuclear periphery, nucleolus, and in nuclear matrix, where they could interact with transcription factors [47, 48]. For example, MUC1-C could activate TCF7L2 transcription factor and promote the expression of cyclinD1, which regulates self-renewal of mammary stems [42]. According to another report, MUC1-C interacts with cytosolic chaperones, such as HSP70 and HSP90, to concentrate in the outer membrane of mitochondria. And it is supposed to be mediated by hereguli/ERBB receptor/c-Src signaling. The MUC1-C mitochondrial localization inhibited the release of cell-death causing factors and blocks stress-induced activation of the intrinsic apoptotic pathway by interfering Bcl-2 subfamily members localization thus neutralization of Bcl-2/Bcl-xL proteins in colon cancer cells [43]. Intriguingly, MUC1-C is illustrated targeted to lysosomes by interacting with HSP70 as well in pancreatic cancer cells, Besides, the researchers observed that inhibition of MUC1 expression leads to an increase in the activity of Cathepsin B in the cytosol, which indicated lysosomal permeabilization, and further promotes cancer cells' death. Therefore, MUC1-C was illustrated to keep pancreatic cancer cells' survival by inhibiting lysosomal permeabilization [49].

## Understanding of transmembrane mucins in lung adenocarcinoma

**Fig. 3:**
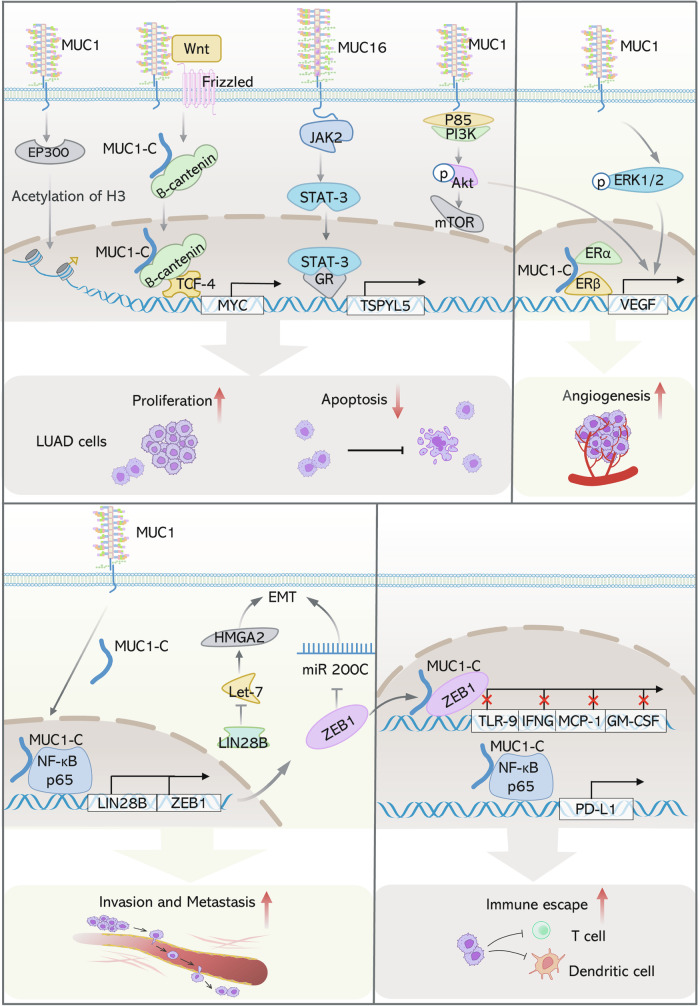
Differential signaling of MUC1 and MUC16 in LUAD pathogenesis. MUC1 stimulates the growth of LUAD cells and prevents their death by overexpressing MYC and turning on the PI3K/AKT/mTOR pathway. MUC16 controlled the development of LUAD cells via the JAK2/STAT3/GR axis. MUC1 activates the ERK and AKT pathways to upregulate VEGF and encourage angiogenesis. MUC1 induces EMT via upregulating the expression of LIN28B and ZEB1, which then promotes LUAD cell invasion and metastasis. MUC1-C interacts with ZEB1 and NFκB/P65 to engage in immunological escape by regulating TLR-9, IFNG, MCP-1, GM-CSF, and PD-L1.

Almost 1.8 million people worldwide lose their lives to lung cancer each year [50]. The LUAD accounts for the major subtype of all histopathological lung cancer types. Multiple studies have reflected that transmembrane mucins are aberrantly expressed and widely involved in lung cancer behaviors [10, 30, 51] (Fig. 3).

### Proliferation and apoptosis

In H1973 cells, blocking MUC1-C by inhibitory peptide led to a decrease of proliferative ability owing to the repression of estradiol–activated reporter gene, endogenous cyclin D1 gene, and nuclear respiratory factor-1 gene transcription [52]. Raina et al. used cell-penetrating peptide as MUC1-C inhibitor to prevent MUC1 from binding to PI3K-p85 in A549 and H1975 cells, leading to suppression of phosphorylating AKT and mTOR. The results substantiated that MUC1 promoted LUAD progression through activating the PI3K/AKT/mTOR pathway [53]. Similarly, silencing MUC1 gene suppressed the phosphorylation of AKT and ERK so as to inhibit proliferation of NCI-H1650 cells [54]. It was reported that targeting MUC1-C would inhibit AKT and MEK signaling in KRAS mutant LUAD cells (A549/KRAS and H460/KRAS cells). Subcutaneous tumor xenograft models generated with A549 and H460 cells stably expressing MUC1-targeting shRNA demonstrated significantly smaller tumor volumes compared to the control groups in nude mice [55]. MUC1 induced histone H3 acetylation and activation of transcription of MYC gene, which has important potential in regulating tumor self-renewal and proliferation via WNT/β-catenin pathway in A549/KRAS and H460/KRAS cells. Additionally, MUC1 inhibition could downregulate CDK4 (a targeting gene of MYC) to weaken the survival and growth of KRAS mutant LUAD [56]. Other reports investigated that MUC1 could interact with c-SRC in A549 paclitaxel‑resistant cells and was involved in proliferation and apoptosis mediated by STAT3 and focal adhesion kinase [57, 58]. Similarly, silence of MUC1 up-regulated Bax and Caspase-3, down-regulated Bcl-2, resulting in inhibition of proliferation and acceleration of apoptosis in A549 paclitaxel‑resistant cells [59]. MUC1 resulted in downregulating the expression of anti-apoptotic protein Bcl-xl, further elevating the apoptosis of the A549 cells. H358 cells transfected with MUC1-targeting siRNA were orthotopically injected into mouse lungs. Compared to control groups transfected with non-targeting siRNA, MUC1 knockdown resulted in observable reductions in tumor weight and volume [60]. Besides, the interaction between dissociative MUC1-C and ER-α, ER-β in the nucleus contributed to inhibiting LUAD cells' proliferation [52]. MUC4 up-regulated p53 led to the accumulation of cells at the G2/M phase of cell cycle progression, it decreased AKT activation and cyclins D1 and E, but increased the expression of p21 and p27, which is the molecular mechanism for MUC4 inhibiting the proliferation of LUAD in H292 cells [61]. For MUC16, Lakshmanan et al. demonstrated that MUC16 regulated A549 cells' growth through TSPYL5 via the JAK2/STAT3/GR axis [62]. In vivo studies showed that subcutaneous H292 cells xenografts with MUC16 knockdown (shMUC16-seq1/seq2) exhibited markedly reduced tumor volume and weight compared to scramble controls at 4 weeks [63].

### Invasion and metastasis

It has been displayed that orthotopic implantation of MUC1-knockdown H358 cells into mouse lungs markedly reduced mediastinal metastasis and pleural effusion incidence compared to non-targeting siRNA controls [60]. Kharbanda et al. silenced MUC1-C and found ZEB1 decreased and miR-200c increased, which provided evidence for the notion that MUC1 was necessary for epithelial-mesenchymal transition (EMT) by driving ZEB1 and miR-200c in A549/KRAS and H460/KRAS cells [55]. It has been certified that miR-200c was an inducer of epithelial differentiation and played a coordinate upregulation role of ZEB1 in the induction of EMT [64]. Furthermore, MUC1-C promoted the uptake of the first intron of LIN28B by NF-κB p65 chromatin and activated LIN28B transcription, driving LIN28B/let-7/HMGA2 signaling to facilitate EMT phenotype and self-renewal of H1975, H1299, and H1650 cells [65]. For MUC16, it was validated to induce LUAD cells' migration through EMT. Knockdown MUC16 resulted in reduced Src phosphorylation, decreased N-cadherin, and increased CK-18 expression, where overexpression of MUC16 rescued the phenotype [63]. On the contrary, researchers revealed in vitro experiments that MUC4 could alter FAK phosphorylation, N-cadherin, and E-cadherin to abrogate the ability of LUAD cells (H292 and A549) to migrate and invade [61]. To further evaluate the role of MUC4 in EMT and metastasis in vivo, researchers established MUC4 knockdown LTEP xenograft models. They observed an increase in vimentin and a decrease in E-cadherin in MUC4-deficient lung tissues versus controls. Additionally, MUC4 expression was lower in metastatic lymph nodes than in primary tumors in patients with LUAD. These in vivo findings are consistent with cellular data supporting MUC4 as a metastasis suppressor [66].

### Tumor angiogenesis

The transmembrane mucins have been established to be involved in angiogenesis to promote invasion and metastasis of lung cancer. MUC1 was represented to be co-expressed with a variety of angiogenic factors and their receptors in NSCLC. The significant co-expression was noted as thymidine phosphorylase, vascular endothelial growth factor (VEGF), VEGF-receptor KDR, basic fibroblast growth factor (bFGF), and bFGF-receptor. These findings indicated that MUC1 significantly participated in angiogenic of NSCLC [62]. Yao et al. verified that the MUC1-induced VEGF upregulation was accomplished by means of the AKT and ERK signaling pathways in A549 and NCI-H460 cells [10]. In an immunohistochemical investigation of 185 LUAD specimens (diameter< or =3 cm), the expression of MUC4 and p27 correlated with blood vessel invasion; besides, MUC4 could be detected mostly in stromal invasion regions [67]. Moreover, MUC16 was illustrated to form aggregates with neutrophils, macrophages, and platelets, providing protection for cancer cells during hematologic dissemination [68].

### Immune surveillance

Transmembrane mucins have been discovered to affect the immune microenvironment of lung cancer through dissimilar molecular biological mechanisms, including influencing immune checkpoints, immune factors.

The reduction or absence of human leukocyte. Antigen I (HLA I) is considered to weaken the immune cells’ function of recognizing and killing cancer cells significantly [69]. There are several studies that suggest that HLA I deletion was a common event in NSCLC, the loss rate could be as high as 93.6% [70]. Koukourakis et al. reported that NSCLC patients exhibiting combined MUC1 overexpression and loss of HLA class I expression were significantly associated with adverse clinical outcomes. The study probably provided proof of MUC1 is involved in the immune reprogramming of lung cancer cells, while the regulatory mechanism between MUC1 and HLA I still requires in-depth study [59]. The blockade of programmed death 1 (PD-1) and programmed death ligand 1 (PD-L1) immune checkpoint, as an effective immunotherapy, has changed the landscape of NSCLC therapy, but with only a 20% response rate [71]. Bouillez et al demonstrated that the expression of PD-L1 depended on MUC1 in both H1975/EGFR and A549/KRAS cells. The mechanism was that MUC1 increased the uptake of NF-κB p65 occupancy on the PD-L1 promoter to drive the transcription of PD-L1. Additionally, in immunocompromised nude mice with H460 xenografts, targeting the MUC1-C cytoplasmic domain suppressed tumor growth and PD-L1 expression, identifying MUC1-C as a therapeutic target for PD-L1 downregulation [64]. Immune-competent MUC1 transgenic mouse models indicated that PD-L1 was upregulated and interferon-γ was inhibited in Lewis Lung Carcinoma cells overexpressing MUC1-C [72].

MUC1 suppresses immune responses by regulating the expression of multiple innate and adaptive immune factors. For example, MUC1 has been revealed to induce activating the NF-κB/ZEB1 pathway, leading to a poor clinical outcome of NSCLC patients, and the process was effected by the suppression of immune factors, such as TLR9, IFNG, MCP-1 (monocyte chemoattractant protein-1), and GM-CSF genes [64]. An analysis of Oncomine database and cBioPortal TCGA data set suggested that the overexpression of MUC1 was negatively correlated with immune factors such as CD8, IFNG, and granzyme B. This was considered to be associated with poor prognosis in NSCLC [72]. Besides, there is evidence that MUC16 has the capability to inhibit innate immune response by regulating NK cells and macrophages [68, 73]. Chen et al. investigated the clinic features and MUC16 expression in NSCLC patients, it turned out that patients with less MUC16 expression had a higher white blood cell count, besides the patients with more MUC16 expression had less white blood cell count, especially neutrophil count [74]. Hence, the result helped to explain the poor prognosis caused by high expression of MUC16.

## Diagnosis, prognosis, and therapy for lung cancer

The specific change of structure, function, and expression level of the transmembrane mucins in NSCLC provides a strong theoretical basis and enormous research potential for clinical application. For example, in patients with NSCLC, MUC1 expression gradually increased from the precancerous lesions (atypical hyperplasia) to invasive carcinoma, indicating that MUC1 may be used as a biomarker in the detection and management of NSCLC [75].

### Diagnostic markers

In a bioinformatics analysis and prospective study done by Arroyo et al. they discovered that among 2819 differentially expressed genes in LUAD. MUC1, CAPN8-2, and TMC5 provided a meaningful diagnostic and prognosis value for LUAD by a high-throughput sequencing of small cohorts of Biobanco samples, since they are activated 2.55 to 6.96 fold in LUAD but repressed in small cell lung cancer and squamous cell carcinoma of the lung [76]. Pan et al. detected purified proteins from exosomes of NCI-H838 cells in contrast to total cellular membrane proteins by semi-quantitative proteomic analysis and found that MUC1 expression in exosomes was 8.98 times that of total cell membrane proteins. Besides they analyzed the levels of MUC1 in plasma exosomes of 27 NSCLC patients and 16 healthy controls, it could be seen that the levels were up-regulated (mean value 1.55 ± 0.16 vs mean value 1.05 ± 0.06, *p* = 0.0213), while the content of MUC1 detected in the plasma in NSCLC was not significantly different from that of healthy controls. This implied that MUC1 in plasma exosomes could be used as a more sensitive and effective diagnostic marker of NSCLC [77]. By comparing the immunohistochemical expression of MUC1, MUC2, MUC5AC and MUC6 in 19 ALK-lung cancers or 42 EGFR-mutated lung cancers, LEE et al. suggested that MUC1presented in the cytoplasm in ALK+ cancer cell, but stained in apical area (92.9%) and focally in cytoplasmic staining (7.1%) in EGFR-mutated cancer cell. The experiment implied that MUC1 expressed in cytoplasmic could be a significant indicator for distinguishing ALK+ from EGFR-mutated lung cancer. Since aberrantly cytoplasmic MUC1 has been demonstrated to be expressed in the proximal, juxtabronchial progenitors of alveolar structures, the expression pattern may imply the origin of ALK+ cancer cells [78]. The MUC1-N and MUC1-C subunit forms a complex that could be shed from the surface of tumor cells and secreted to the outside of cells, so it can be detected in plasma or pleural fluid [17]. As proof, the investigators established a proteomics pipeline to verify potential biomarkers (MUC1, ALCAM, CDH1, SPINT1, and THBS4, AUC > 0.7) and another panel (SPINT1/SVEP1/THBS4, AUC = 0.95), which effectively discriminated lung cancer malignant pleural effusion from benign [79]. MUC4 was substantially overexpressed in LUAD, not expressed in malignant mesothelioma and benign mesenchymal cells, thus distinguishing LUAD from malignant mesothelioma with 100% specificity and 91.4% sensitivity by immunohistochemical staining [80]. In a prospective clinical study of 63 ever-smoking lung cancer patients and 90 matched controls, researchers developed an integrated lung cancer risk prediction model that combined smoking exposure with a panel of MUC16, CEA, CYFRA 21-1, and pro-SFTPB in peripheral blood. Its sensitivity and specificity were both higher than those of the single smoking model (sensitivity: 0.63 to 0.43; specificity: 0.95 to 0.86) [81]. In a study conducted to evaluate the diagnostic markers MUC16 in bronchoalveolar lavage fluid (BALF) and serum, investigators observed that MUC16 concentration was higher in 46 lung cancer patients than in 42 healthy volunteers, besides the BALF had a significantly higher value for the individual markers than that of serum. The specificity of MUC16 in BAL and serum was 75% to 62.5% [82]. With a carbohydrate content of up to 24%, MUC16 flows more easily into local bronchoalveolar space, after which it enters the blood circulation to be diluted [83]. Therefore, mucins, as tumor markers, appeared earlier and at higher concentrations in the bronchoalveolar fluid than in serum in early-stage lung cancer. It has a significant clinical value in detecting mucins in BALF with more supersensitive and quantitative methods.

### Prognosis markers

Differential expression levels of a variety of transmembrane mucins have been investigated as significant prognosis-related indicators of LUAD. The RNA sequencing profile from the public database represented that the mRNA expressions of MUC3A, MUC4, MUC13, MUC16, and MUC21 were significantly up-regulated in LUAD patients; the up-level mRNA of MUC1 and MUC15 were significantly associated with a favorable overall survival (OS), especially in LUAD patients [84]. Researchers investigated different expressions and calculated the final immune reactivity score of MUC1, MUC2, MUC5AC, and MUC6 in 99 LUAD resection specimens, and among them, the IRS of MUC1 > 8.5(*p* = 0.018) made it a valuable predictor for lymph node metastasis [85]. Zhu et al. certified that patients with positive MUC1 mRNA in circulating tumor cells (CTCs) had a significant lower disease-free survival and OS before and after surgery (*p* = 0.01 before and *p* = 0.002 after surgery), which certified MUC1 mRNA as a useful biomarker of CTCs to assist in the prognosis of LUAD [86].

Lung cancer cells have various simple and short sugar chain structures of MUC1-N terminal, which are different from normal cells. This alteration of glycosylation patterns in lung cancer cells generates several epitopes in the oligosaccharide side chains, such as Tn antigen (GalNAcα1‑O‑Ser/Thr, CD175). Medical scientists obtained that LUAD with more MUC1-Tn had a higher consolidation/tumor ratios, more intake of 18F-fluorodeoxyglucose, leading poor survival of LUAD patients [17]. TA-MUC1 is a conformationally distinct MUC1 glycoform defined by tumor-specific carbohydrate modifications. The investigators used the monoclonal antibody PankoMab (anti-TA-MUC1) to perform immunohistochemistry on 85 NSCLC patients and found that PankoMab staining was significantly associated with patient survival (*p* = 0.029), and patients with lymph node metastases lacking PankoMab staining had the highest risk of death. This study provides the first evidence of a pro-survival MUC1 glycoform, highlighting TA-MUC1 emerged as an independent prognostic marker [87]. The function of MUC1 is also reflected in detecting the efficacy of antineoplastic agents as a prospective indicator. For instance, in a clinical trial of 66 advanced NSCLC patients treated with gefitinib (a drug for EGFR-TKI), investigators detected the expression of MUC1 and VEGF mRNA before gefitinib treatment (B0) and 4 weeks after that (B4w) by real-time fluorescence quantitative PCR. The results validated that the mRNA level increased significantly at both time points above (B0 and B4w) and was closely associated with shorter progression-free survival (PFS) and OS. It revealed that the positivity mRNA of MUC1 and VEGF had become an indicator of the poor efficacy of patients with advanced NSCLC receiving gefitinib [88]. Krebs Von den Lungen-6 (KL-6) is a glycoform of MUC1 with 5000 kDa and classified as “cluster 9 (MUC1)” based on carbohydrate composition analysis [89]. The association between serum KL-6 levels and NSCLC survival in a 103-cohort was validated by Tanaka et al. They discovered that elevated circulating KL-6 levels were linked to poor PFS and OS (*p* = 0.041 and 0.023, respectively) with multivariate analysis of the independent prognostic value [90]. Ishikawa et al. supported the above conclusion through monitored circulating KL-6 levels in 74 NSCLC patients treated by EGFR-TKI and observed that the levels were higher in patients with progressive disease (PD) than in patients with disease control [partial remission (PR) and stable disease (SD)]. Changes in KL-6 levels 2 weeks after initiation of EGFR-TKI therapy could accurately predict the clinical outcome of PD, PR, and SD patients of NSCLC [91]. In a study of clinical stage III-IV 277 NSCLC (including 107 LUAD) patients, the serum secretion MUC16 level in LUAD patients was higher than those of other histological types and the elevated level predicted poor prognosis, compared to patients with normal CA125, PFS and OS were: 4.6 vs. 7.5 months, *p* < 0.05, and 8.7 vs. 14.0 months, *p* < 0.05, respectively [92]. The concentration of MUC16 and NSE in the serum was significantly associated with liver metastasis of NSCLC patients. Combination both of the two resulted in sensitivity (51.2%), specificity (72.6%), and area under the curve (0.64) values, which were higher than single factor so as to provide a useful forecasting method for liver metastasis [93].

### Therapy adequate

Over the past decade, targeted therapy and immunotherapy combined with other therapy regimens hold the promise of improving the survival of NSCLC patients. Transmembrane MUCs are also involved in these two concepts. Currently, in clinical antitumor treatment, mucin-based therapies are mainly divided into two types, mucin-targeted drugs and immunotherapy (vaccine, chimeric antigen receptor (CAR)-T cell therapy, etc.) (Table 1). At present, there are multiple MUC1-targeting drugs under intense development.

**Table 1:** Therapeutic Schedule of NSCLC.

Treatment type	Method	Target	Ref
Mucins-targeted drugs	Combine GSTA neoantigen-specific 16A with MMAE as ADC	MUC1	[94]
	Combine evodiamine with anti-PD-1 monoclonal antibody	MUC1	[95]
	Combine MUC-1/CD3-BsAb and EpCAM/CD3-BsAb	MUC1	[96]
	MUC1 aptamer functionalized hybrid nanoparticle targeted drug delivery systems	MUC1	[97–99]
	GO-203	MUC1	[100]
Cancer vaccines	L-BLP25	MUC1	[103]
	TG4010	MUC1 and interleukin 2	[104]
CAR-T	CV9202 DC-based vaccines MUC1-targeted CAR T cells	NY-ESO-1, MAGEC1, MAGEC2, 5 T4, survivin, and MUC1 MUC1 MUC1	[105] [106] [107]

This table outlines current therapeutic approaches targeting transmembrane mucins in NSCLC, categorized by treatment type and method, with corresponding molecular targets and key references.

#### Mucins-targeted drugs

The use of antibody-drug conjugates (ADC) as efficient substitutes has increased since antibodies to abnormally glycosylated proteins are ineffective. GSTA is a specific glycosylated neoantigen peptide located in the tandem repeat region of abnormal glycosylation of MUC1-N. Pharmaceutical scientists combined GSTA neoantigen-specific 16A with monomethyl auristatin E (MMAE) to synthesize anti-MUC1 ADC, of which showed strong anti-tumor activity in NSCLC [94]. Furthermore, Jiang et al. investigated that the combination of evodiamine and anti-PD-1 monoclonal antibody in the treatment of NSCLC was achieved through the targeted downregulation of MUC1-C/PD-L1 axis and the increase of CD8+ T cells [95]. Bispecific antibody (BsAb) increases the affinity and specificity of targeted therapy by targeting two different antigens. Based on this, experts developed MUC-1/CD3-BsAb and EpCAM/CD3-BsAb, the combination of the two significantly suppressed tumor growth in A549, H1975, H466 cells and corresponding mouse models, because of the enhancement of cell toxic lymphocyte, an increase of type I interferon and T cells number in tumor-draining lymph nodes [96]. Owing to technological advances in biomaterials, more and more MUC1 aptamer functionalized hybrid nanoparticle targeted drug delivery systems have been successfully developed, such as PLA-PEG-Apt/DOX NPs, MUC-1 peptide-loaded non-aggregated poly (lactide-co-glycolide) nanoparticles, miRNA-29b loaded aptamer functionalized nanoparticles. These new biologics possessed better accuracy and stability so as to inhibit the activity of NSCLC cells by anti-tumor proliferation, increasing apoptosis, and they held a broad clinical application prospect [97–99]. Besides, the CQC motif of MUC1-C was capable of binding with L- and D-amino acid CQCRRK-containing peptides named GO-203. It would block homodimerization of MUC1 in LUAD cells so as to provide a potential drug target [100].

#### Immunotherapy

Commonly, NSCLC surface antigens developed as vaccines include MAGE A3, MUC1, EGF, and TGF-β [101]. Nowadays, the improved survival of Phase I/II clinical trials targeting the MUC1 tumor antigen provides a proof of further larger phase III trials, especially in combination with chemoradiotherapy or some targeted therapies [102]. L-BLP25 (Stimuvax®/Tecemotide) liposome vaccine consists of immunogenic peptide of MUC1 (BLP25 lipopeptide) and other lipid components. In a randomized Phase IIb study of L-BLP25 in stage IIIB and IV NSCLC, patients received L-BLP25 plus best supportive care (BSC) had a median survival time of 17.2 months and a 3-year survival rate of 31%, compared to 13.0 months and a 3-year survival rate of 17% in patients receiving BSC alone. It offered strong evidence that vaccines made contributed to improving NSCLC patients’ survival rate [103]. Other than L-BLP25, TG4010 is an antigenic vaccine based on a poxvirus that codes for MUC1 tumor-associated antigen and interleukin 2, the follow-up evolution of TG4010 and chemotherapeutics for 6 months in 148 patients of advanced (stage IIIB or IV) NSCLC expressing MUC1 showed that the combination treatment regimen got a higher PFS than that chemotherapy alone. As a result, TG4010 enhanced the chemotherapy effect of advanced NSCLC patients in the phase IIB study, and the phase 2B-3 validation tests have been initiated [104]. CV9202 is a self-adjuvating mRNA vaccine that targets six antigens (NY-ESO-1, MAGEC1, MAGEC2, 5 T4, survivin, and MUC1) expressed in NSCLC, inducing a broad antigen immune response simultaneously. In a clinical trial (NCT01915524), NSCLC patients of stage IV and those with responsive or stable disease after first-line chemotherapy or EGFR tyrosine kinase inhibitors participated in the evaluation of the CV9202’s safety and tolerability combined with local radiation designed to enhance the immune response. Current findings supported the feasibility of their combination therapy [105]. Dendritic cell (DC)-based vaccines targeting MUC1 have been demonstrated to increase NSCLC patients’ survival in MUC1-positive cases. who do not respond to standard anticancer therapies. DCs loaded with MUC1-derived peptides induced antitumor immune responses, the occurrence of immune-related adverse events was substantiated to be a beneficial immunotherapy feedback, besides a higher percentage of peripheral lymphocytes was a biomarker predicting good efficacy [106].

CAR-T cell therapy has also emerged in the treatment of NSCLC in recent years. Wei et al. verified that MUC1-targeted CAR-T cells effectively inhibited the growth of NSCLC cancer cells in patient-derived xenograft mouse models, and they acted synergistically with prostate stem cell antigen-targeted CAR-T cells. The result implies that the targeting of antigen combinations is conducive to enhancing the efficacy of CAR-T cells [107]. Tn-MUC1 has been identified as a strategic CAR-T target due to its ubiquity across pan-cancer malignancies. Tmunity Therapeutics’ Tn-MUC1-targeted CAR-T therapy (NCT04025216) is currently in Phase I trials, with the dose-escalation cohort enrolling patients with Tn-MUC1-high tumors, including NSCLC, ovarian cancer, pancreatic cancer, and triple-negative breast cancer [108].

## Conclusion and prospectives

Over the past three decades, hundreds of studies have been conducted on mucins as biomarkers and targets in various types of cancers [109]. Significant advancements have been achieved in understanding the function of transmembrane mucins (mainly MUC1, MUC4, and MUC16) in NSCLC progression, such as proliferation, invasion, metastasis, and immune escape. It should be mentioned that when studying NSCLC in the past, some researchers did not differentiate between squamous cell carcinoma and adenocarcinoma. The findings of such research may not be applicable to squamous cell carcinoma, and more studies are needed to further confirm. It has been established that MUC1 is strongly expressed in a number of malignancies, including prostate, ovarian, lung, and breast cancer. Numerous MUC1-targeted drugs have been developed and have shown promising therapeutic outcomes in clinical trials. Drug development of other mucins in lung cancer is still in its infancy since their molecular structures and regulatory mechanisms are not well understood. Further study of the differential distribution, specific domain function, special epitopes formed by abnormal glycosylation modification, presentation of intracellular downstream signals, and the ways to induce cell-to-cell/microenvironment interactions would assist in exposing more potential targets for clinical research.

Since the concentration of mucins in BALF is generally higher than that in the serum of lung cancer patients, the detection of sensitive mucins as markers in BALF could lead to a breakthrough in the diagnosis of early lung cancer. Besides, it is meaningful to evaluate the relationship between changes in different mucin levels and patients’ survival and response to medication therapy. Improving the accuracy of evaluating the correlation would provide evidence for therapeutic regimen adjustment. Nevertheless, there are critical and practical issues that need to be addressed in the future. Remodeling the immunotherapeutic microenvironment, thus amplifying the efficacy of transmembrane mucins-based vaccines, is worth exploring. How to enhance efficacy and reduce toxicity to maximize the effect of therapy is also a question. Clinical evidence needs to be accumulated substantially to confirm which combination regimen offers the best therapeutic impact. In general, owing to the rapid biological technology, we have seen great application prospects for transmembrane mucins in NSCLC and have confidence in holding a vision for a better future.
